# Arsenic-Transformed Malignant Prostate Epithelia Can Convert Noncontiguous Normal Stem Cells into an Oncogenic Phenotype

**DOI:** 10.1289/ehp.1204987

**Published:** 2012-04-04

**Authors:** Yuanyuan Xu, Erik J. Tokar, Yang Sun, Michael P. Waalkes

**Affiliations:** National Toxicology Program Laboratory, Division of the National Toxicology Program, National Institute of Environmental Health Sciences, National Institutes of Health, Department of Health and Human Services, Research Triangle Park, North Carolina, USA

**Keywords:** arsenic, cancer stem cells, interluekin-6, prostate, stem cells

## Abstract

Background: Cancer stem cells (CSCs) are likely critical to carcinogenesis, and, like normal stem cells (NSCs), are affected by microenvironmental factors. Malignant cells release extracellular factors, modifying tumor behavior. Inorganic arsenic, a human carcinogen, is associated with an overproduction of CSCs in various model systems of carcinogenesis.

Objective: We aimed to determine if NSCs are influenced by nearby arsenic-transformed malignant epithelial cells (MECs) as a possible factor in arsenic-associated CSC overabundance.

Methods: Transwell noncontact co-culture allowed the study of the effects of non-contiguous, arsenic-transformed prostate MECs on the isogenic human prostate NSC line, WPE-stem. Cancer phenotype was assessed by evaluating secreted matrix metalloproteinases (MMPs), invasiveness, colony formation, and spheroid formation. Gene expression was assessed at the protein (Western blot) or mRNA (real-time reverse transcription–polymerase chain reaction) levels.

Results: Noncontact co-culture of MECs and NSCs rapidly (≤ 3 weeks) caused hypersecretion of MMPs and marked suppression of the tumor suppressor gene *PTEN* in NSCs. NSCs co-cultured with MECs also showed increased invasiveness and clonogenicity and formed more free-floating spheroids and highly branched ductal-like structures in Matrigel, all typical for CSCs. MEC co-culture caused dysregulated self-renewal and differentiation-related gene expression patterns and epithelial-to-mesenchymal transition in NSCs consistent with an acquired cancer phenotype. Interleukin-6 (IL-6), a cytokine involved in tumor microenvironment control, was hypersecreted by MECs, and IL-6 exposure of NSCs resulted in the duplication of several responses in NSCs of conversion to CSCs via MEC co-culture (e.g., MMP hypersecretion, decreased *PTEN*).

Conclusions: Arsenic-transformed MECs recruit nearby NSCs into a cancer phenotype, thereby potentially increasing CSC number. This may be a factor in arsenic-induced CSC overabundance seen in multiple model systems.

Cancer stem cells (CSCs) are generally considered to be a minor tumor subpopulation but the driving force for tumor initiation, progression, and metastasis (Rosen and Jordan 2009). Initially detected in leukemia (Bonnet and Dick 1997), CSCs have now been identified in other tumors and cell transformants based on surface markers, phenotypic traits indicative of malignancy (such as xenograft tumor formation), and *in vitro* characteristics including matrix metalloproteinase (MMP) secretion, colony formation, and formation of nonadherent spheroids. (Tokar et al. 2010a; Visvader and Lindeman 2008). CSCs share characteristics with normal stem cells (NSCs), although CSCs show dysregulated self-renewal (Bomken et al. 2010). The origin of CSCs and the mechanisms involved in their formation are incompletely defined. Recent studies indicate that CSCs may be derived from transformed NSCs (Iliopoulos et al. 2010; Wang 2010), although committed progenitors or even differentiated cells also may produce CSCs (Wang 2010).

Inorganic arsenic threatens millions of persons through contaminated drinking water [International Agency for Research on Cancer (IARC) 2011]. Arsenic is a human carcinogen (IARC 2011), and although underlying mechanisms remain uncertain, it appears capable of both genotoxic and epigenetic mechanisms of initiation (Kojima et al. 2009). The prostate is considered a possible target of arsenic carcinogenesis (IARC 2011). In mice, inorganic arsenic causes tumor development in the urogenital system (Waalkes et al. 2007). Accumulating evidence suggests that stem cells (SCs) likely play a key role in arsenic carcinogenesis. For example, human epidermal cells exposed to inorganic arsenic maintain a germinative state with altered SC dynamics (Patterson et al. 2005). Chronic inorganic arsenic *in vitro* causes malignant transformation of the human prostate epithelial cell line, RWPE-1 (also termed CAsE-PE cells) (Achanzar et al. 2002), and can similarly induce a malignant phenotype in an isogenic prostate NSC line, WPE-stem (Tokar et al. 2010a). Arsenic-transformed prostate NSCs rapidly produce highly aggressive, immature and pluripotent tumors in mice, qualifying the transformed NSCs as CSCs (Tokar et al. 2010a). Furthermore, when the whole population of prostate epithelial cells is malignantly transformed by arsenic, it involves a survival selection for, and overproduction of, CSCs (Tokar et al. 2010b). Similarly, in a mouse carcinogenesis model, prenatal arsenic exposure distorts postnatal skin SC dynamics, increases chemically induced formation of squamous cell carcinoma, and increases tumor CSC content relative to tumors induced without prenatal arsenic exposure (Waalkes et al. 2008). Interestingly, it has long been appreciated that arsenic-induced cases of human skin cancer are typically multifocal (IARC 1973), potentially endowing multiple cells with tumor-forming capacity (i.e., CSCs). Further, human skin keratinocytes malignantly transformed by inorganic arsenic *in vitro* show a clear overabundance of CSCs, even compared to transformation by effective skin carcinogens like UV-irradiation (Sun et al. 2012). Likewise, arsenic-induced lung and liver tumors in mice show a clear CSC overabundance compared with spontaneous tumors and cancers induced by organic carcinogens (Tokar et al. 2011a). Thus, both *in vivo* and *in vitro* data indicate that inorganic arsenic produces a consistent CSC overabundance at the same time it causes acquisition of malignant phenotype.

Like NSCs, CSCs typically reside in a microenvironmental niche (Borovski et al. 2011). Many microenvironmental factors influence tumor growth by modifying responses at the SC level. For instance, the growth potential of prostate CSCs is enhanced by their production of inflammatory cytokines including interluekin-6 (IL-6) (Liao et al. 2010), which can stimulate or regulate CSCs through a variety of pathways (Korkaya et al. 2011). Thus, the dynamic equilibrium between CSCs and more “differentiated” tumor cells is thought to be mediated, at least in part, by the microenvironment, including local cytokine secretion (Iliopoulos et al. 2010; Korkaya et al. 2011; Liao et al. 2010).

Since malignant cells release various soluble factors that modify tumor behavior, some of which also regulate CSCs (Iliopoulos et al. 2010; Korkaya et al. 2011; Liao et al. 2010), we suspected that malignant epithelial cells may affect non-contiguous NSCs. We hypothesized that this could be a way in which arsenic induces overproduction of CSCs in tumors or induces multifocal tumors. Thus, using the prostate normal stem/progenitor cell line WPE-stem (henceforth referred to as NSCs) we assessed the impact of neighboring arsenic-transformed malignant epithelial cells (MECs) on NSCs via noncontact co-culture. Our results indicate that the prostate MECs drive nearby NSCs into a cancer phenotype, in effect creating putative CSCs without any actual physical contact. This tumor “recruitment” of NSCs potentially constitutes a new phenomenon in cancer growth and dissemination, and could help account for the repeated observations of arsenic-induced CSC overabundance in tumors or malignantly transformed cells.

## Materials and Methods

*Cell lines and culture.* All cell lines used in the present study are isogenic. The normal human prostatic epithelial cell line, RWPE-1, a gift from M. Webber (Michigan State University), is heterogeneous, containing stem/progenitor, intermediate, and differentiated cell types (Tokar et al. 2005). The WPE-stem cell line (NSCs) was isolated from the RWPE-1 line by single-cell dilution cloning and shows characteristics of urogenital system stem/progenitor cells, with anchorage-independent growth, nonadherent spheroid formation, high expression of *p63*, *K5*, *K14*, *ABCG2*, and *BMI*, and low expression of *K8*, *K18*, *AR*, and *PSA* (Tokar et al. 2005, 2010a). These NSCs also show self-renewal as evidenced by serial sphere passage capacity (data not shown). Arsenic malignantly transformed prostate epithelia (originally termed CAsE-PE cells; in this study termed MECs) were developed from RWPE-1 cells chronically exposed to sodium arsenite (5 μM) and show multiple malignant phenotype characteristics, including hypersecretion of MMP-9, colony formation in agar, and aggressive xenograft malignancies (Achanzar et al. 2002; Tokar et al. 2010b).

To investigate potential effects of neighboring cells on NSCs, NSCs were exposed to MECs or control RWPE-1 cells via a co-culture system (Corning Life Sciences, Suwanee, GA) that did not allow direct contact between the two cell types. NSCs were seeded into the lower compartment of 6-well transwell plates. After 1 day to allow NSCs to attach, malignant or control epithelial cells were seeded on the transwell inserts (collagen-coated 0.4-µm pore PTFE (polytetrafluoroethylene) membrane). The cell layers were approximately 1 mm apart in this system. The MECs were derived as reported by Achanzar et al. (2002) and used for exposure to NSCs in the co-culture system. The MECs (about 3 million cells) were first washed twice with 5 mL Dulbecco’s phosphate-buffered saline (DPBS) and lifted using 2 mL trypsin and 4 mL DPBS before adding in the insert (75,000 cells/insert). Direct evaluation of conditioned medium from MECs during co-culture (at 3 weeks) revealed no detectable arsenic above background by atomic absorption spectrophotometry.

Cell culture surfaces for SC were coated with type IV collagen (Trevigen, Gaithersburg, MD) and fibronectin (BD Biosciences, Bedford, MD) (2.5 μg of each/mL). Cells were maintained in low-calcium, serum-free medium [keratinocyte serum-free medium (KSFM)] containing 50 μg/mL bovine pituitary extract, 5 ng/mL epidermal growth factor, and 1% antibiotic-antimycotic mixture (all from Gibco, Rockville, MD), to maintain the stem/progenitor cell phenotypic status (Litvinov et al. 2006). Cells were incubated at 37°C in 5% CO_2_ with a medium change every 48 hr and passaged weekly. Various assessments were carried out weekly during 3 weeks of co-culture. Sphere assessment continued for an additional week, as it took 1 week for the spheres to form.

*MMP activity.* Secreted MMP activity is highly correlated with malignant transformation in the cells used in this study (Achanzar et al. 2002; Tokar et al. 2010a, 2010b). After co-culture with MECs, SC were re-plated and grown alone to collect conditioned medium, and secreted MMP-9 or MMP-2 activity was examined by zymography (Tokar et al. 2005).

*Free-floating sphere formation.* The formation of free-floating spheres of viable cells is characteristic of NSCs and CSCs (Tokar et al. 2005, 2010b). Weekly during MEC co-culture, SC were collected by trypsin, filtered through a 40-µm cell strainer (BD Falcon, Franklin Lakes, NJ) to a single cell suspension, and plated in uncoated 6-well plates. Cells were fed every 48 hr. After 1 week, floating spheres and adherent cells were collected separately, and viable cells were quantitated by automated cell counter.

*Anchorage-independent growth.* Colony formation in soft agar from cells derived from free-floating spheres is related to the NSC or CSC phenotype (Stingl et al. 2006) and is indicative of anchorage-independent growth. After noncontact co-culture, floating spheroid cells were separated from adherent cells, and colony formation in soft agar was assessed (Tokar et al. 2005).

*Branched ductal-like structures and serial passage.* After 3 weeks of co-culture, single SC were seeded into uncoated 6-well plates until free-floating spheres formed. Free-floating spheres were collected, dissociated by pipette trituration and filtered through a 40-µm cell strainer. Cells were suspended in 1:1 Matrigel/KSFM growth medium in a total volume of 200 µL, plated in a 24-well plate, and placed in the incubator overnight. The next day, 1 mL of KSFM growth medium was added on top of the solidified Matrigel mixture. The medium was changed every 3 days. Photomicrographs were taken with an inverted microscope after 2 weeks. For serial passage (a measurement of self-renewal capacity typical of NSCs or CSCs), colonies in Matrigel were collected, disassociated into single cells, and re-plated into 24-well plates in the Matrigel mixture as described above. The formation of new colonies in Matrigel was accomplished at least 3 times.

*Invasion.* Invasive ability as a malignant phenotype was assessed using a modified Boyden chamber assay (Bello et al. 1997).

*Assessment of gene expression.* Transcript levels of *p63, WNT3*, *OCT*-*4*, *NOTCH-1*, *K5*, *K18*, *PTEN,* and *E-CAD* were examined by real-time reverse transcription–polymerase chain reaction (RT-PCR) as described previously (Tokar et al. 2010a). Cycle time (Ct) values were normalized based on control = 100% using the average values of β-actin (*ACTB*) and glyceraldehyde 3-phosphate dehydrogenase (*GAPDH*) from the same sample. Primer sequences are provided in Supplemental Material, Table 1 (http://dx.doi.org/10.1289/ehp.1204987).

For Western blots, protein was collected with M-PER Mammalian Protein Extraction Reagent (Pierce, Rockford, IL), containing 0.1 mM phenylmethyl sulfonyl fluoride (Sigma-Aldrich, St. Louis, MO) and 1% Protease Inhibitor Cocktail (Thermo Scientific, Rockford, IL). Western blots were performed as described previously (Tokar et al. 2010b). Protein bands were assessed by ECL Reagent (GE Healthcare, Buckinghamshire, UK).

*Immunofluorescence.* NSCs co-cultured with MECs were washed with DPBS and fixed with acetone and methanol (1:1, vol:vol). For staining, cells were blocked with normal horse serum (room temperature, 1 hr), incubated with primary antibodies [rabbit anti-K5 (ABcam, Cambridge, MA), mouse anti-p63 (Santa Cruz Biotechnology, Santa Cruz, CA) or mouse anti-VIMENTIN (Sigma-Aldrich)], at 4°C, overnight, rinsed, incubated at room temperature for 1 hr with secondary antibodies labeled with Alexa Fluor 488 or 568 (Molecular Probes, Eugene, OR), and rinsed again. Cells were also stained with DAPI (4´,6-diamidino-2-phenylindole dihydrochloride) (Invitrogen, Eugene, OR) at room temperature for 10 min. After washing with DPBS, photomicrographs were captured with an automated Olympus inverted fluorescence microscope, equipped with a 40× objective (Olympus Corporation, Center Valley, PA). Photomicrographs were processed by CellSens software (Olympus Corporation) with consistent settings (e.g., exposure time) for both control and MEC groups.

*IL-6 assessment and treatment.* Using normal culture conditions, IL-6 secreted into culture medium by MECs over 24 hr was determined using the human IL-6 ELISA kit (R&D Systems, Minneapolis, MN). NSCs treated with 20 ng/mL IL-6 for 1 week were analyzed for characteristics indicative of cancer phenotype such as MMP-9 activity and expression of *K5*, *p63*, *PTEN*, *E-CADHERIN* (*E-CAD*) and *VIMENTIN*.

*Statistical analysis.* Data represent means and standard errors (SE). Student’s *t*-test was used in all comparisons, with a *p* < 0.05 considered significant.

## Results

**Figure 1:**
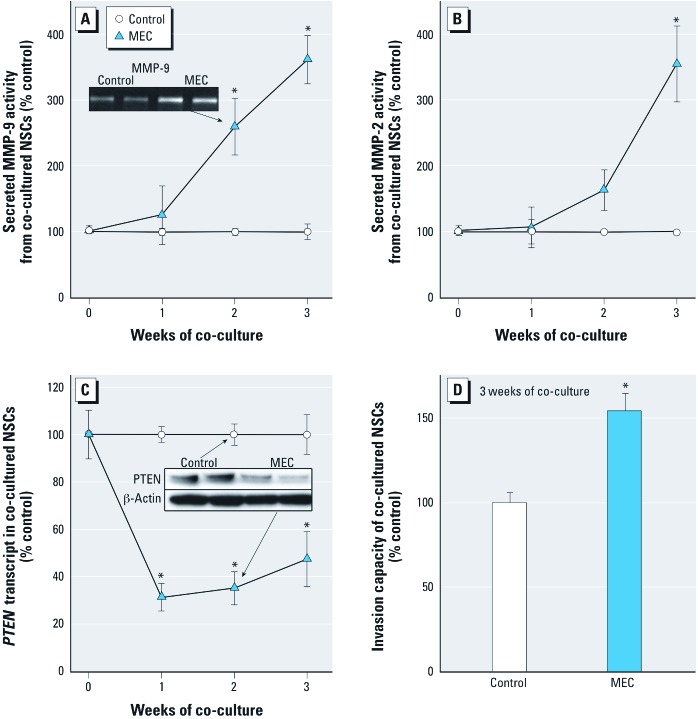
Secreted MMP activity, *PTEN* expression and invasiveness of NSCs during noncontact co-culture with prostate MECs or control RWPE-1 cells. (*A*) MMP-9 activity of NSCs during co-culture. Inset: representative zymogram of secreted MMP-9 at 2 weeks. (*B*) MMP-2 activity of NSCs during co-culture. (*C*) Expression of *PTEN* in NSCs during co-culture. Inset: example of PTEN protein levels determined by Western blot at 2 weeks. (*D*) Invasion capacity of NSCs co-cultured after 3 weeks. Quantitative results are presented as mean ± SE; *n* = 3 except for invasion where *n* = 7. *p < 0.05, compared with time-matched control.

*Noncontact MEC co-culture and NSCs transformation.* Secreted MMP activity is strongly associated with malignant transformation for the cells used in this study. Indeed, after only 2 weeks of noncontact co-culture with MECs, NSCs showed hypersecretion of MMP-9, and by 3 weeks also showed hypersecretion of MMP-2 (Figure 1A, B). NCSs co-cultured with MECs showed maximal secreted MMP-9 activity of 3.6 times control and maximal MMP-2 activity of 3.5 times control. Cells isogenic with the NSCs and MECs used in this work showing increased secreted MMP-9 activity in the range of 2.0 to 4.5 times control consistently produce malignant tumors upon inoculation into mice (Achanzar et al. 2002; Tokar et al. 2010b). *PTEN,* a tumor suppressor gene that plays a critical role in SC differentiation and is often inactivated in malignancies, was rapidly and persistently suppressed in NSCs with MEC co-culture at both the transcriptional (Figure 1C) and translational (Figure 1C inset) levels. Invasive capacity in NSCs also increased after MEC co-culture (Figure 1D).

**Figure 2:**
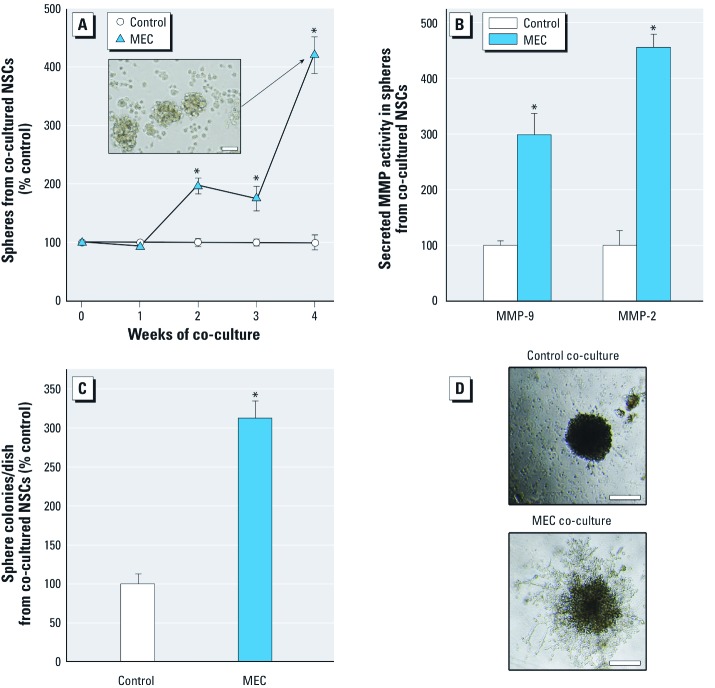
Assessment of acquired cancer phenotype in NSCs noncontact co-cultured with MECs or control RWPE-1 cells. (*A*) Viable floating nonadherent spheroids from NSCs after co-culture. Inset: free-floating spheres formed after MEC co-culture (bar = 50 μm). (*B*) Secreted MMP-9 and MMP-2 activity in nonadherent spheroids formed after co-culture. (*C*) Colony formation from spheres in soft agar after co-culture. (*D*) Ductal-like structure formed by single cell from the floating sphere produced after co-culture. Structures formed in Matrigel over 2 weeks (bar = 200 μm). Data are presented as mean ± SE (*n* = 3). *p < 0.05, compared with time-matched control.

Nonadherent spheroid-forming ability is a common characteristic of NSCs and CSCs. Thus, sphere-forming capacity and the CSC-like phenotype of free-floating spheres were assessed after noncontact co-culture. By 4 weeks, the number of viable free-floating spheres produced by SCs after MEC co-culture was 4 times the number produced after control co-culture (Figure 2A). Secreted MMP activity of the spheroid SCs formed by noncontact MEC co-culture also markedly increased relative to the MMP activity of spheroid SCs formed after co-culture with control cells (Figure 2B). Colony formation of spheroid cells in soft agar, an indication of anchorage-independent growth common to cancer cells, was markedly increased in spheroid cells produced by MEC (vs. control) co-culture (Figure 2C). After 3 weeks of MEC co-culture, single SCs formed spheres with a highly branched ductal-like structure in Matrigel (Figure 2D; bottom) indicative of an aggressive phenotype. The spheres formed in Matrigel from NSCs co-cultured with control cells showed minimal branching (Figure 2D; top). Cells from the structures formed in Matrigel after MEC co-culture could be serial passaged (≥ 3 times), indicative of the self-renewal capacity typical of CSCs.

**Figure 3:**
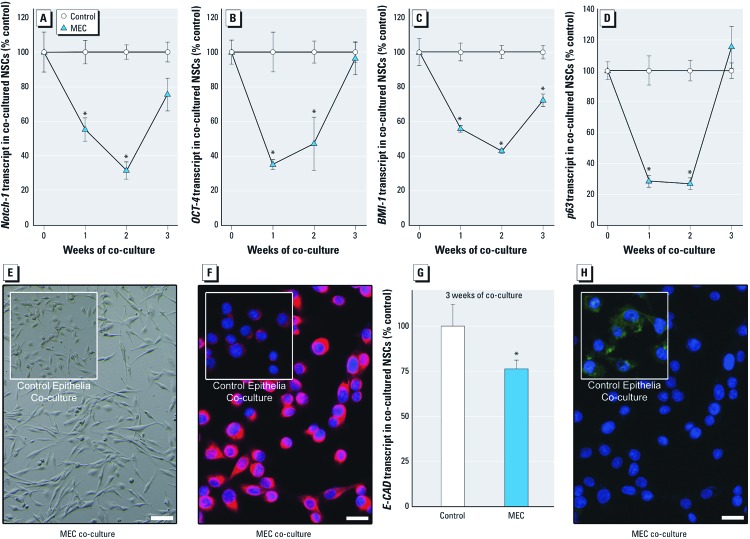
Expression of genes associated with SC self-renewal and maintenance, and signs of EMT after noncontact co-culture with MECs or control RWPE-1 cells. Transcript levels of *NOTCH-1* (*A*), *OCT-4* (*B*), *BMI-1* (*C*), and *p63* (*D*). The morphologic alteration of NSCs (bar = 200 μm; *E*), the hyper-expression of *VIMENTIN* (bar = 20 μm; *F*), and the decrease in *E-CAD* transcript (*G*), and protein expression (bar = 20 μm; *H*) indicative of EMT of SCs after co-culture with MECs for 3 weeks. NSCs after co-culture with control epithelial cells are shown in insets for (*E*) and (*F*) for comparison. Results are mean ± SE (*n* = 3). *p < 0.05, compared with time-matched control.

**Figure 4:**
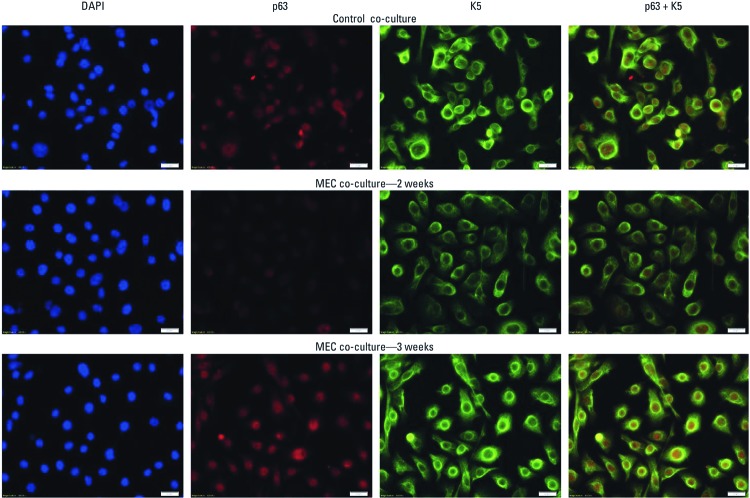
Fluorescence microscopic imaging of protein expression levels of K5 (green) and p63 (red) in NSCs after noncontact co-culture with MECs or control RWPE-1 cells for 2 or 3 weeks (bar = 20 μm). Both K5 and p63 decreased after 2 weeks of co-culture with MECs, but returned to control levels after 3 weeks. Control protein levels are shown for 2 weeks of exposure of NSCs to control epithelial cells and show similar expression at 0, 1, or 3 weeks. DAPI (blue) is a general nuclear stain used to indicate cell number.

*SC-related gene expression and epithelial-to-mesenchymal transition (EMT) with co-culture.* The prostate SC-associated genes *NOTCH-1*, *OCT-4, BMI-1*, and *p63* all showed an early transcription expression loss in SCs during the first 2 weeks of noncontact co-culture with MECs (Figure 3A–3D). By 3 weeks of MEC co-culture, these genes were reactivated in SCs to levels at or near control. WNT signaling activation, which is considered a functional marker of CSCs (Vermeulen et al. 2010), showed a similar “U-shaped” expression pattern in SCs over 3 weeks of MEC co-culture [see Supplemental Material, Figure 1A (http://dx.doi.org/10.1289/ehp.1204987)]. In addition, *K5*, a marker of undifferentiated prostate SCs, also showed loss followed by reactivation in SCs co-cultured with MECs (see Supplemental Material, Figure 1B). *K18*, a marker of cellular differentiation showed an opposite expression trend (see Supplemental Material, Figure 1C). EMT of SCs occurs as early stage tumors are converted into invasive cancers, and is a critical mechanism for acquisition of metastatic capacity (Kang and Massague 2004). After 3 weeks of MEC co-culture, SCs exhibited depolarized spindle-like morphology (Figure 3E), increased *VIMENTIN* expression (Figure 3F) and decreased *E-CAD* expression (Figure 3G and 3H), all indicative of EMT. Normally, the NSCs used in this study show homogeneous K5 and p63 expression. When NSCs were co-cultured with MECs, K5 and p63 protein levels decreased homogeneously across all cells by 2 weeks, but increased and returned to control levels by 3 weeks (Figure 4), consistent with the patterns observed for transcript levels (Figure 3 and Supplemental Material, Figure 1). These results were qualitatively confirmed by Western blot (not shown).

**Figure 5:**
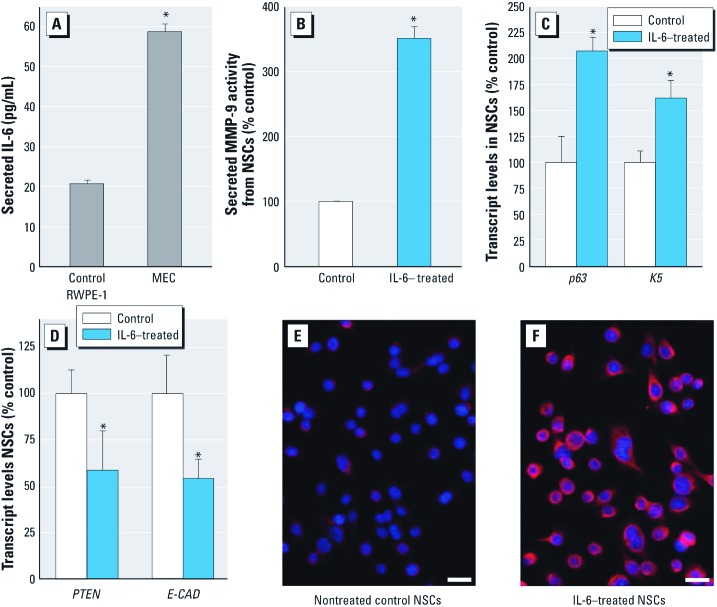
Secretion of IL-6 by MECs and the transforming effects of IL-6 (20 ng/mL for 1 week) on NSCs. (*A*) IL-6 secreted by control RWPE-1 cells and MECs over 24 hr. (*B*–*D*) Effects of exposure to IL-6 on NSCs vs. nontreated control NSCs shown by secreted MMP-9 activity (*B*), transcript levels of *K5* and *p63* (*C*), and transcript levels of *PTEN* and *E-CAD *(*D*)*. *Results for *A–D* represent mean ± SE (*n* = 3 or 4). (*E*) *VIMENTIN* expression in control NSCs. (*F*) *VIMENTIN* expression in NSCs after exposure to IL-6. For *E* and *F*, bar = 20 μm. *p < 0.05, compared with nontreated control.

*IL-6 and acquired cancer phenotype in NSCs.* Over a 24-hour period using normal culture conditions, MECs cultured alone secreted 2.8 times more IL-6 than control epithelial cells (Figure 5A). Furthermore, after only 1 week of IL-6 treatment, NSCs showed a 350% increase in MMP-9 secretion relative to controls (Figure 5B), and a marked increase in *p63* and *K5* expression (Figure 5C), while *PTEN* and *E-CAD* expression decreased (Figure 5D). The marked elevation of *VIMENTIN* expression in IL-6-treated NSCs compared with control (Figure 5E and 5F) provides molecular evidence of EMT after IL-6 exposure. These results indicate NSCs rapidly acquire a cancer-like phenotype after IL-6 exposure that is similar in nature to the phenotype acquired after NSCs are co-cultured with MECs.

## Discussion

Arsenic is a human carcinogen, and the prostate is a potential target organ (IARC 2011). We have shown direct arsenic exposure transforms human prostate NSCs into CSCs more rapidly (18 weeks) (Tokar et al. 2010a) than their heterogeneous parental epithelial prostate cells (RWPE-1 cells) are transformed into MECs (29 weeks) (Achanzar et al. 2002). In the present study, the NSC line was very rapidly pushed towards a CSC-like phenotype by nearby MECs previously transformed by arsenic. Temporally, this is consistent with CSCs being capable of initiating malignant growth. Because the co-culture system did not contain arsenic or allow physical contact between NSCs and MECs, this transformation was likely due to factors secreted by the MECs. The distance between the MECs and NSCs in this co-culture system (1 mm) is comparable to the width of about 100 prostate epithelial cells. This phenomenon may constitute a novel “recruitment” by arsenic-produced malignant prostate epithelial cells of nearby prostate NSCs into putative CSCs that could affect tumor growth, invasion, dissemination, or field cancerization. As such, this suggests a potentially important aspect of arsenic carcinogenesis that may help explain why arsenic causes CSC overabundance in various *in vivo* and *in vitro* model systems of carcinogenesis (Achanzar et al. 2002; Sun et al. 2012; Tokar et al. 2010b, 2011b; Waalkes et al. 2008). It will be very interesting to see if MECs originally transformed by other carcinogens can similarly recruit NSCs into CSCs.

The *PTEN* tumor suppressor gene is frequently mutated, deleted, or inactivated in prostate cancers (Verhagen et al. 2006). During MEC co-culture, SC *PTEN* was rapidly and persistently suppressed as the oncogenic phenotype was acquired. *PTEN* inactivation appears important for the early selection and expansion of SCs during malignant transformation (Verhagen et al. 2006). The loss of PTEN activity enhances cell proliferation, decreases apoptosis, and leads to the expansion of prostatic CSC-like cells, tumor initiation, and metastasis (Wang et al. 2006). For prostate SCs, *PTEN* knockout increases CSC-like sphere formation capacity and tumorigenic potential (Dubrovska et al. 2009). Thus, the suppression of *PTEN* expression in this study strongly supports the notion that prostatic SCs were transformed to a CSC-like phenotype by MEC co-culture.

Dysregulated self-renewal programming of CSCs is typical during oncogenesis (Pardal et al. 2003). During MEC co-culture, an early loss and subsequent reactivation of various SC self-renewal genes, such as *NOTCH-1*, *OCT-4*, *BMI-1*, *p63*, and *WNT3* occurred. *NOTCH-1* is a prostate SC marker that can act as both an oncogene and tumor suppressor gene in tumors (Leong and Gao 2008). *OCT-4* regulates self-renewal in SCs, and is potentially involved in the maintenance of CSC-like properties (Hochedlinger et al. 2005). *BMI-1* is crucial to both NSCs and CSCs (Lessard and Sauvageau 2003) and, in prostate cancer, is an essential regulator of SC renewal and tumor initiation and progression (Lukacs et al. 2010). The WNT pathway regulates NSC self-renewal, and hyperactivation is linked to carcinogenesis (Vermeulen et al. 2010). *p63* is required for self-renewal and proliferative capacity of prostate SCs (Senoo et al. 2007) and promotes neoplastic growth (Nylander et al. 2002). The time-related, decreased-then-increased, gene expression pattern during acquisition of oncogenic characteristics in SCs seen in our study is consistent with the expression of SC-related genes observed in NSCs during arsenic-induced malignant transformation (Tokar et al. 2010a). Indeed, when NSCs were malignantly transformed by direct arsenic exposure, the expression of various critical SC self-renewal and differentiation genes was first lost and then regained as cells acquired a malignant phenotype (Tokar et al. 2010a). This pattern of SC-related gene response is also consistent with that observed during transformation of hematopoietic SCs into leukemic SCs (Krivtsov et al. 2006; Somervaille and Cleary 2006). Inappropriate activation of the embryonic SC-like transcriptional program appears to induce CSCs in adult epithelial cells (Wong et al. 2008). Thus, the distorted expression of SC-related genes observed in this study is likely an important feature of CSC formation. In addition, *K5* and *K18*, markers for undifferentiated and differentiated epithelia, respectively (Schalken and van Leenders 2003), showed opposing expression patterns (i.e., *K5,* loss and regain; *K18,* increase and then loss), fortifying the notion of initial dysregulation of SC characteristics during “recruitment” and subsequent transformation by nearby MECs into CSCs.

The fate of CSCs is dynamically orchestrated by their microenvironment (Liao et al. 2010; Suzuki et al. 2011). Secreted extracellular matrix degradation factors, growth factors, and cytokines can facilitate tumor progression and enhance malignant characteristics of CSCs (Liao et al. 2010; Suzuki et al. 2011). IL-6, a pleiotrophic inflammatory cytokine, is elevated in the serum of prostate cancer patients and related to progression and metastases in prostate cancers (Azevedo et al. 2011). *In vitro* IL-6 induces malignant conversion of benign prostate epithelia (Rojas et al. 2011), although the role of SCs in this process is unknown. In breast cancer cell lines, IL-6 converts non-stem cancer cells into CSCs (Iliopoulos et al. 2010). Although many agents cause IL-6 to be secreted, the overexpression or hypersecretion of IL-6 can be induced by arsenic exposure both *in vivo* (Das et al. 2005) and *in vitro* (Lee et al. 2005). In the present work, prostate MECs under normal culture conditions secreted high levels of IL-6, consistent with stimulation of autocrine IL-6 loops, conferring oncogenic properties (Rojas et al. 2011). Furthermore, direct IL-6 treatment of NSCs led to oncogenic alterations (e.g., MMP hypersecretion, loss of PTEN) similar to those seen when NSCs underwent noncontact MEC co-culture. These results suggest that IL-6 may facilitate transformation of NSCs to CSCs during noncontact MEC co-culture.

## Conclusions

These data support the concept that NSCs can be driven towards a CSC phenotype as a key aspect of arsenic-induced oncogenesis. Furthermore, at least with prostate cells, “recruitment” appears to occur where arsenic-transformed MECs send out signals, potentially including IL-6, which can convert NSCs into CSC-like cells over a significant distance. This recruitment by cancer cells of NSCs into putative CSCs may facilitate tumor growth and dissemination and may help explain the repeated observation of CSC overabundance induced by arsenic in model systems of carcinogenesis.

## Supplemental Material

(119 KB) PDF

## References

[r1] Achanzar WE, Brambila EM, Diwan BA, Webber MM, Waalkes MP (2002). Inorganic arsenite-induced malignant transformation of human prostate epithelial cells.. J Natl Cancer Inst.

[r2] Azevedo A, Cunha V, Teixeira AL, Medeiros R (2011). IL-6/IL-6R as a potential key signaling pathway in prostate cancer development.. World J Clin Oncol.

[r3] Bello D, Webber MM, Kleinman HK, Wartinger DD, Rhim JS (1997). Androgen responsive adult human prostatic epithelial cell lines immortalized by human papillomavirus 18.. Carcinogenesis.

[r4] Bomken S, Fiser K, Heidenreich O, Vormoor J. (2010). Understanding the cancer stem cell.. Br J Cancer.

[r5] Bonnet D, Dick JE (1997). Human acute myeloid leukemia is organized as a hierarchy that originates from a primitive hematopoietic cell.. Nat Med.

[r6] Borovski T, De Sousa EMF, Vermeulen L, Medema JP (2011). Cancer stem cell niche: the place to be.. Cancer Res.

[r7] Das S, Santra A, Lahiri S, Guha Mazumder DN (2005). Implications of oxidative stress and hepatic cytokine (TNF-α and IL-6) response in the pathogenesis of hepatic collagenesis in chronic arsenic toxicity.. Toxicol Appl Pharmacol.

[r8] Dubrovska A, Kim S, Salamone RJ, Walker JR, Maira SM, Garcia-Echeverria C (2009). The role of PTEN/AKT/PI3K signaling in the maintenance and viability of prostate cancer stem-like cell populations.. Proc Natl Acad Sci USA.

[r9] Hochedlinger K, Yamada Y, Beard C, Jaenisch R. (2005). Ectopic expression of OCT-4 blocks progenitor-cell differentiation and causes dysplasia in epithelial tissues.. Cell.

[r10] IARC (International Agency for Research on Cancer) (1973). Arsenic and inorganic compounds.. IARC Monogr Eval Carcinog Risk Hum.

[r11] IARC (International Agency for Research on Cancer) (2011). Arsenic and arsenic compounds.. IARC Monogr Eval Carcinog Risk Hum.

[r12] Iliopoulos D, Hirsch HA, Wang G, Struhl K (2010). Inducible formation of breast cancer stem cells and their dynamic equilibrium with non-stem cancer cells via IL6 secretion.. Proc Natl Acad Sci USA.

[r13] Kang Y, Massague J. (2004). Epithelial-mesenchymal transitions: Twist in development and metastasis.. Cell.

[r14] Kojima C, Ramirez DC, Tokar EJ, Himeno S, Drobna Z, Styblo M (2009). Requirement of arsenic biomethylation for oxidative DNA damage.. J Natl Cancer Inst.

[r15] Korkaya H, Liu S, Wicha MS (2011). Regulation of cancer stem cells by cytokine networks: Attacking cancer’s inflammatory roots.. Clin Can Res.

[r16] Krivtsov AV, Twomey D, Feng Z, Stubbs MC, Wang Y, Faber J (2006). Transformation from committed progenitor to leukaemia stem cell initiated by MLL-AF9.. Nature.

[r17] Lee P-C, Ho I-C, Lee T-C (2005). Oxidative stress mediates sodium arsenite-induced expression of heme oxygenase-1, monocyte chemoattractant protein-1, and interleukin-6 in vascular smooth muscle cells.. Toxicol Sci.

[r18] Leong KG, Gao WQ (2008). The notch pathway in prostate development and cancer.. Differentiation.

[r19] Lessard J, Sauvageau G. (2003). *Bmi-1* determines the proliferative capacity of normal and leukaemic stem cells.. Nature.

[r20] Liao CP, Adisetiyo H, Liang M, Roy-Burman P (2010). Cancer-associated fibroblasts enhance the gland-forming capability of prostate cancer stem cells.. Cancer Res.

[r21] Litvinov IV, Vander Griend DJ, Xu Y, Antony L, Dalrymple SL, Isaacs JT (2006). Low-calcium serum-free defined medium selects for growth of normal prostatic epithelial stem cells.. Cancer Res.

[r22] Lukacs RU, Memarzadeh S, Wu H, Witte ON (2010). Bmi-1 is a crucial regulator of prostate stem cell self-renewal and malignant transformation.. Cell Stem Cell.

[r23] Nylander K, Vojtesek B, Nenutil R, Lindgren B, Roos G, Zhanxiang W (2002). Differential expression of p63 isoforms in normal tissues and neoplastic cells.. J Pathol.

[r24] Pardal R, Clarke MF, Morrison SJ (2003). Applying the principles of stem-cell biology to cancer.. Nat Rev Cancer.

[r25] Patterson TJ, Reznikova TV, Phillips MA, Rice RH (2005). Arsenite maintains germinative state in cultured human epidermal cells.. Toxicol Appl Pharmacol.

[r26] Rojas A, Liu G, Coleman I, Nelson PS, Zhang M, Dash R (2011). IL-6 promotes prostate tumorigenesis and progression through autocrine cross-activation of IFG-IR.. Oncogene.

[r27] Rosen JM, Jordan CT (2009). The increasing complexity of the cancer stem cell paradigm.. Science.

[r28] Schalken JA, van Leenders G (2003). Cellular and molecular biology of the prostate: Stem cell biology.. Urology.

[r29] Senoo M, Pinto F, Crum CP, McKeon F (2007). p63 is essential for the proliferative potential of stem cells in stratified epithelia.. Cell.

[r30] Somervaille TC, Cleary ML (2006). Identification and characterization of leukemia stem cells in murine MLL-AF9 acute myeloid leukemia.. Cancer Cell.

[r31] Stingl J, Eirew P, Ricketson I, Shackleton M, Vaillant F, Choi D (2006). Purification and unique properties of mammary epithelial stem cells.. Nature.

[r32] Sun Y, Tokar EJ, Waalkes MP (2012). Overabundance of putative cancer stem cells in human skin keratinocyte cells malignantly transformed by arsenic.. Toxicol Sci.

[r33] Suzuki K, Sun R, Origuchi M, Kanehira M, Takahata T, Itoh J (2011). Mesenchymal stromal cells promote tumor growth through the enhancement of neovascularization.. Mol Med.

[r34] Tokar EJ, Ancrile BB, Cunha GR, Webber MM (2005). Stem/progenitor and intermediate cell types and the origin of human prostate cancer.. Differentiation.

[r35] Tokar EJ, Diwan BA, Waalkes MP (2010a). Arsenic exposure transforms human epithelial stem/progenitor cells into a cancer stem-like phenotype.. Environ Health Perspect.

[r36] Tokar EJ, Diwan BA, Ward JM, Delker DA, Waalkes MP (2011a). Carcinogenic effects of “whole-life” exposure to inorganic arsenic in CD1 mice.. Toxicol Sci.

[r37] Tokar EJ, Qu W, Liu J, Liu W, Webber MM, Phang JM (2010b). Arsenic-specific stem cell selection during malignant transformation.. J Natl Cancer Inst.

[r38] Tokar EJ Qu W Waalkes MP 2011b Arsenic, stem cells, and the developmental basis of adult cancer. Toxicol Sci 120 Suppl 1:S192 S203 21071725 10.1093/toxsci/kfq342 PMC3043086

[r39] Verhagen PC, van Duijn PW, Hermans KG, Looijenga LH, van Gurp RJ, Stoop H (2006). The PTEN gene in locally progressive prostate cancer is preferentially inactivated by bi-allelic gene deletion.. J Pathol.

[r40] Vermeulen L, De Sousa EMF, van der Heijden M, Cameron K, de Jong JH, Borovski T (2010). Wnt activity defines colon cancer stem cells and is regulated by the microenvironment.. Nat Cell Biol.

[r41] Visvader JE, Lindeman GJ (2008). Cancer stem cells in solid tumours: Accumulating evidence and unresolved questions.. Nat Rev Cancer.

[r42] Waalkes MP, Liu J, Diwan BA (2007). Transplacental arsenic carcinogenesis in mice.. Toxicol Appl Pharmacol.

[r43] Waalkes MP, Liu J, Germolec DR, Trempus CS, Cannon RE, Tokar EJ (2008). Arsenic exposure in utero exacerbates skin cancer response in adulthood with contemporaneous distortion of tumor stem cell dynamics.. Cancer Res.

[r44] Wang JC (2010). Good cells gone bad: the cellular origins of cancer.. Trends Mol Med.

[r45] Wang S, Garcia AJ, Wu M, Lawson DA, Witte ON, Wu H (2006). *Pten* deletion leads to the expansion of a prostatic stem/progenitor cell subpopulation and tumor initiation.. Proc Natl Acad Sci USA.

[r46] Wong DJ, Liu H, Ridky TW, Cassarino D, Segal E, Chang HY (2008). Module map of stem cell genes guides creation of epithelial cancer stem cells.. Cell Stem Cell.

