# Supplementary material for: Arsenic-Transformed Malignant Prostate Epithelia Can Convert Noncontiguous Normal Stem Cells into an Oncogenic Phenotype
Source: Environ Health Perspect. 2012 Apr 4;120(6):865–71. doi: 10.1289/ehp.1204987 (PMC3385457; doi:10.1289/ehp.1204987)
Supplement: (119 KB) PDF [file ehp.1204987.s001.pdf]

Supplemental Material

Arsenic-Transformed Malignant Prostate Epithelia Can Convert Noncontiguous Normal Stem Cells into an Oncogenic Phenotype

Yuanyuan Xu, Erik J. Tokar, Yang Sun and Michael P. Waalkes

Table of Contents

Page	Number	Content
2	Supplemental Material, Table 1	Genes and Primers for real time RT-PCR
3	Supplemental Material, Figure 1	Transcript level expression of WNT3 (A), K5 (B) and K18 (C) in NSCs after non-contact co-culture with MECs (Malignant Epithelial Cells)

Supplemental Material, Table 1 Genes and Primers for real time RT-PCR

Gene	GenBank Accession No.	Primers (5’→ 3’)
P63 (Tumor protein 63)	NM-003722	Forward: CCCCAAGCAGTGCCTCTACA<br>Reverse: GGTGAATCGCACAGCATCAA
NOTCH-1 (Notch homolog 1)	NM_017617	Forward: CGGGTCCACCAGTTTGAATG<br>Reverse: GTTGTATTGGTTCGGCACCAT
OCT4/POU5F1 (OCTomer 4/POU class 5 homeobox 1)	NM_002701	Forward: CCCCATTTCACCACACTCTACTC<br>Reverse: CCAGAGCAGTGACAGGAACAGA
WNT3 (Wingless-type MMTV integration site family, member 3)	NM_030753	Forward: GCCTGGTCCCCAAGCAA<br>Reverse: GCTGGGCATGATCTCGATGT
K5 (Keratin 5)	NM_000424	Forward: GTAGCAGCTCCAGCGTCAAAT<br>Reverse: TTGGAAGGCAGTGACTTGCA
K18 (Keratin 18)	NM_26326	Forward: GCCTTGGACAGCAGCAACTC<br>Reverse: GACACCACTTTGCCATCCACTA
PTEN (Phosphatase and tensin homolog)	NM_000314	Forward: TTCACATCCTACCCCTTTGCA<br>Reverse: TCTGAGCATTCCCTCCATTCC
E-CAD (E-cadherin)	Z18923	Forward: CGGTGGTCAAAGAGCCCTTAC<br>Reverse: CTCCGCCTCCTTCTTCATCATA

All primers are from Sigma-Aldrich Corp., The Woodlands, TX.

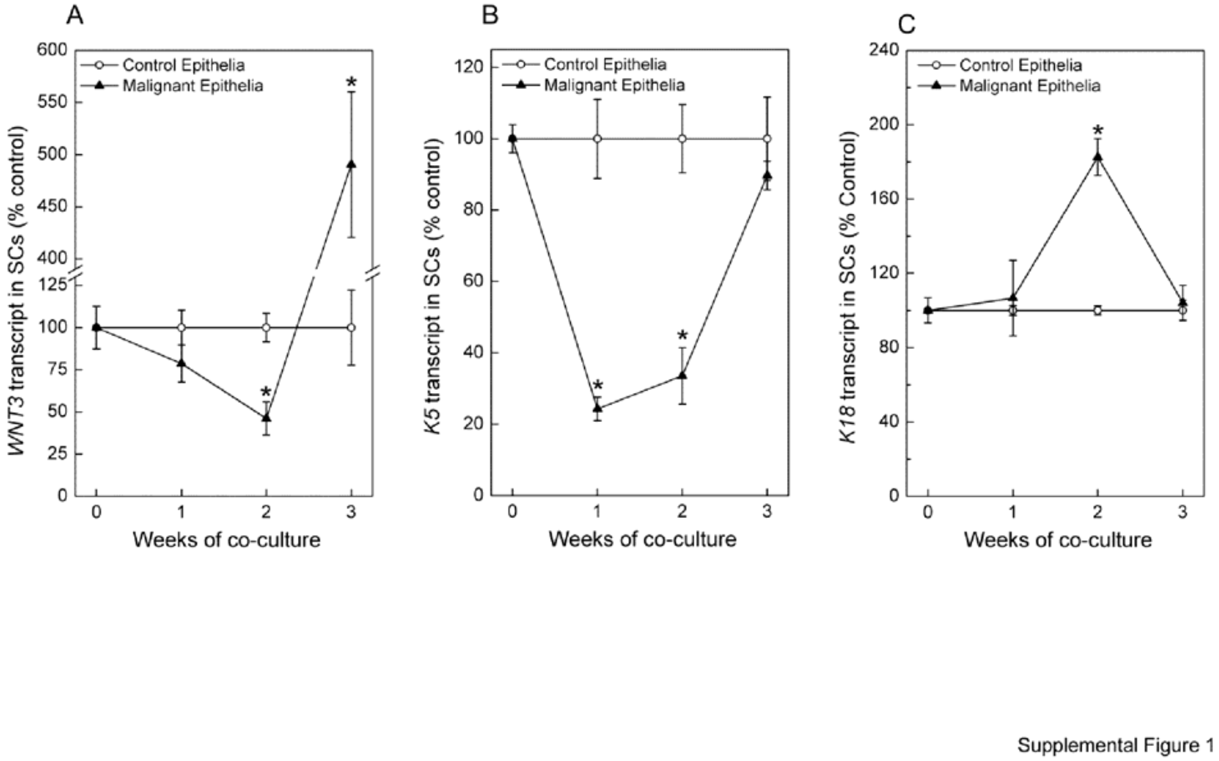

Supplemental Material, Figure 1 Transcript level expression of WNT3 (A), K5 (B) and K18 (C) in NSCs after non-contact co-culture with MECs (Malignant Epithelial Cells). Transcript Levels of K18 showed an early increase of expression then a decrease, just opposite to the change of transcript levels of undifferentiated marker K5. Quantitative data are presented as mean ± SEM, n = 3. * $P < 0.05$, compared with time-matched control.
